# Downregulation of miRNA‐141 in breast cancer cells is associated with cell migration and invasion: involvement of ANP32E targeting

**DOI:** 10.1002/cam4.1024

**Published:** 2017-02-21

**Authors:** Ping Li, Tao Xu, Xin Zhou, Liangying Liao, Guolian Pang, Wan Luo, Lu Han, Jiankun Zhang, Xianyong Luo, Xiaobing Xie, Kuichun Zhu

**Affiliations:** ^1^ Medical Laboratory CenterFirst Affiliated HospitalHunan University of Chinese Medicine95 Shaoshan Middle RoadChangsha410007China; ^2^ Department of Emergency MedicineFirst Affiliated HospitalHunan University of Chinese Medicine95 Shaoshan Middle RoadChangsha410007China; ^3^ Center for Gene DiagnosisZhongnan Hospital of Wuhan University169 Donghu RoadWuhan430071China; ^4^ Department of Scientific ResearchFirst Affiliated HospitalHunan University of Chinese Medicine95 Shaoshan Middle RoadChangsha410007China; ^5^ Department of PathologyFirst People's Hospital of Qujing1 Yuanlin RoadQujing655000China; ^6^ Labway Clinical LaboratoriesShanghai200000China

**Keywords:** *ANP32E*, breast cancer, invasion, migration, miR‐141

## Abstract

MicroRNAs (miRNAs) regulate many cellular activities, including cancer development, progression, and metastasis. Some miRNAs are involved in breast cancer (BC) migration and invasion, thus affect patients’ prognosis. Microarray analysis was performed to compare miRNA expression in BC tissues, and results confirmed by qPCR. BC cell migration and invasion were studied in vitro with MDA‐MB‐231 cells using microplate transwell assays. miRNA targeting was investigated using luciferase assays, qPCR, and Western blot analysis in cells with overexpression of miRNA mimics. Knockdown of miRNA targets was performed using target siRNA lentiviral infection. Results show that microRNA‐141 (miR‐141) was downregulated in breast cancer tumor tissues compared with matched surrounding tissues. Downregulation of miR‐141 expression correlated with tumor stage, lymph node involvement, and expressions of PCNA, Ki67, and HER2. Overexpression of miR‐141 inhibited BC cell proliferation, migration, and invasion in vitro. *ANP32E* gene was selected as one putative target for further studies based on results from in silico analysis. Results from a dual‐luciferase reporter system suggested *ANP32E* as a direct target of miR‐141. Overexpression of miR‐141 downregulated ANP32E expression at both mRNA and protein levels in BC cells. Knockdown of *ANP32E* inhibited BC cell proliferation, migration, and invasion in vitro, mimicking the effect of the overexpression of miR‐141. Our study revealed important roles miR‐141 plays in BC growth and metastasis. Moreover, for the first time, we identified *ANP32E* as one of the miR‐141 targets, and demonstrated its involvement in the regulation of cell proliferation, migration, and invasion.

## Introduction

Breast cancer (BC) is the most frequently diagnosed cancer and the leading cause of cancer death in females that affected an estimated three million women worldwide in 2008 1. About half the breast cancer cases and 60% of the deaths are estimated to occur in developing countries, and most of the deaths are due to metastasis. Although decades of research have provided considerable insight into the multistep metastatic process, the molecular basis of BC metastasis is poorly understood. Thus, further elucidation of the molecular mechanisms of BC metastasis is critical for developing novel therapeutic approaches and the successful management of BC patients.

MicroRNAs (miRNAs), a class of small noncoding RNAs, have been shown to play roles in many aspects of cancer biology, including proliferation, apoptosis, invasion/metastasis, and angiogenesis. The miRNAs, which bind to the 3′‐UTR of their target mRNAs, are proposed to regulate the expression of at least 30% of all protein‐coding genes 2. miRNAs can act either as oncogenes or as tumor suppressors depending on their target mRNAs. Numerous studies have shown that abnormal miRNA expression was related to cancer invasion, metastasis, and resistance to chemotherapy. Thus, miRNA expression profiles are regarded as predictive and prognostic biomarkers for BC 3, 4. miRNAs, such as miR‐21, miR‐210, were overexpressed in BC and the expression levels correlated with tumor invasion and poor prognosis 5. The expression of miR‐200 family members, including miR‐200a, miR‐200b, miR‐200c, miR‐141, and miR‐429, was lost in invasive BC cell lines 6. In BC cells, miR‐141 targets *EGFR*, and inhibits its translation 7. miR‐141 was also found to regulate cancer cell growth and metastasis in lung cancer, liver cancer, colorectal cancer, gastric cancer, renal cancer, and prostate cancer 8, 9, 10, 11, 12, 13.

To screen for miRNAs that play a role in the metastasis process of BC, we conducted miRNA microarray analysis of tissues from BC patients. We found that miR‐141 was downregulated in BC, and miR‐141 expression level negatively correlated with clinicopathologic features such as expressions of Ki67, HER2, and PCNA. miR‐141 overexpression inhibited BC cell proliferation, migration, and invasion in vitro, at least in part through targeting of *ANP32E* (acidic nuclear phosphoprotein 32 kilodalton e). Our work revealed important roles miR‐141 plays in BC development and progression. Both miR‐141 and *ANP32E* might be valuable biomarkers in clinical settings as well as novel therapeutic targets.

## Materials and Methods

### Patients, tumor tissues, and pathology examination

Formalin‐fixed paraffin‐embedded (FFPE) breast tissues (including 106 BC tissues, and 66 nonmalignant tissues paired to BC were collected from the tissue repository in the Zhongnan Hospital of Wuhan University from January 2011 to May 2013. Diagnosis of BC was made following the National Guideline to the Diagnosis and Treatment of Primary Breast Cancer, 2013, Ministry of Health, China. Pathological classification, grading, and staging were made based on WHO Classification of Breast Tumors, 2012, differentiation status of cancer cells, and TNM system 14. Nonmalignant tissues were collected 2 cm away from cancer tissue and microscopically verified to be free of cancer cells. All the FFPE tissues were evaluated histologically by two certified pathologists. BC specimen were classified into subtypes as luminal A‐like, luminal B‐like, HER‐2 overexpression, and Basal‐like according to the expression status of estrogen receptor (ER), progesterone receptor (PR), HER‐2, and Ki67. Each FFPE tissue block was cut into sections of 20 *μ*m in thickness and collected in a 2 mL RNase‐free tube for nucleic acid extraction.

None of the patients received preoperative treatment such as radiotherapy or chemotherapy. All patients were female, and aged 30–79, with an average age at 52.7. In 81% of the cases the tumor size was 2 cm in diameter or larger, and about half of the cases were ER negative and half were PR negative. Tumors at grade I, II, III stages accounted for 61%, 25%, and 13%, respectively. Other clinicopathologic parameters were summarized in Table 1. The study was approved by the Ethics Committee of Zhongnan Hospital of Wuhan University. Informed consents were obtained from all subjects.

**Table 1:** Correlation of miR‐141 expression and clinicopathologic parameters

Clinicopathologic parameters	Cases (%)	miR‐141 expression	*P*‐value
Tumor stage			
T1	53 (50%)	0.024 ± 0.126	0.002
T2	36 (34%)	−0.594 ± 0.142	
T3	10 (9%)	−0.450 ± 0.278	
T4	7 (7%)	−1.036 ± 0.777	
Lymph node metastasis			
No	54 (51%)	−0.122 ± 0.109	0.011
Yes	52 (49%)	−0.581 ± 0.140	
HER‐2 expression			
Negative	43 (41%)	0.009 ± 0.126	0.001
Positive	63 (59%)	−0.591 ± 0.117	
PCNA expression			
Negative	26 (25%)	0.101 ± 0.188	0.025
Positive	80 (75%)	−0.475 ± 0.130	
Ki67 expression			
Negative	31 (29%)	0.324 ± 0.231	0.001
Positive	75 (71%)	−0.499 ± 0.123	

### miRNA microarray assays

Microarray assays were performed in two independent experiments. In the first experiment six BC specimen of Luminal A&B subtype, and with the expressions of ER, PR, and HER‐2 all positive (“Lum”), were used, together with six paired nonmalignant surrounding tissues. In the second experiment, six “Lum” tissue specimen and six BC specimen of Basal‐like subtype, with expressions of ER, PR, and HER‐2 all negative (“Bas”), were used. Microarray assays were performed with the Affymetrix miRNA 3.0 Technology platform. Sample preparation, hybridization, washing, staining, and scanning were performed following the manufacturer's instructions. Expression Console software (version 1.3.1, Affymetrix) was used to analyze array images to get raw data and normalization. GeneSpring software (version 12.5, Agilent Technologies) was used for the subsequent data analysis. The stringent thresholds set for up‐ and downregulated genes were a fold change ≥2.0 and a *P*‐value ≤ 0.05.

### Cell culture, transfection, proliferation, and apoptosis assays

MDA‐MB‐231 cells were obtained from ATCC and cultured in Dulbecco's Modified Eagle Medium (DMEM, GIBICO BRL) with 15% fetal bovine serum (FBS). Cells were seeded in 96‐well plates at 3 × 10^5^ cells/mL in a volume of 100 *μ*L per well and transfected with miR‐141 mimics or NC mimics. MiR‐141 mimics and negative control (NC) mimics were synthesized by GenePharma (Shanghai, China), and were transfected into MDA‐MB‐231 cells at 100 nmol/L by Lipofectamine^TM^ 2000 (Invitrogen) according to the manufacturer's instruction. CCK‐8 assays were performed at 24 h intervals for 5 days after transfection to monitor cell proliferation. Apoptosis was measured 48 h after transfection by PI and Annexin V‐FITC double staining. Flow cytometry analysis was performed using Becton‐Dickinson FACS‐420 flow cytometry according to the protocols recommended by the manufacturer. All experiments were performed in triplicates and repeated three times.

### Transwell migration and invasion assays

Cell migration was evaluated by wound healing assays. MDA‐MB‐231 cells were transfected with miR‐141 mimics or NC mimics, and when cells reached 90% confluence, wound streaks were created by manually scratching the cell monolayer with a 200‐microliter pipette tip. Cells migration into the wound was observed at three preselected time points (0, 24, and 36 h) in eight randomly selected microscopic fields for each condition and time point. Cell images were analyzed with Photoshop software. The widths of the remaining gaps after cell growth for different time intervals were measured, and relative cell migration was calculated by setting the widths of the gaps of NC groups at 0 h as 100%. A wider gap is considered as lower cell migration.

Transwell invasion assays were performed to evaluate cell invasion capability. Transfected MDA‐MB‐231 cells were starved in serum‐free medium for two hours, detached, and resuspended in medium containing 2.5% fetal bovine serum at a density of 4 × 10^5^ cells/mL. The cell suspension (500 *μ*L) was added to the upper chamber of the transwell inserts precoated with matrigel (BD Biosciences). Medium containing 10% fetal bovine serum (750 *μ*L) was added into the bottom of wells of 24 well plates to act as a chemo‐attractant. After an 8‐h invasion period, noninvasive cells in the upper chamber were removed with cotton swabs, and the cells on the lower surface of the inserts were fixed and stained. The numbers of invasive cells were calculated by counting cells in five different fields under microscope from three independent inserts.

For *ANP32E* knocked down cells, cell migration was performed using transwell plates similar to invasion assays but without matrigel coating.

### RNA extraction and qRT‐PCR

Total RNA was extracted from FFPE breast tissue using miRNeasy FFPE Kit (Qiagen) according to the manufacturer's instructions. The concentration and purity of all RNA samples were detected by NanoDrop 2000 spectrophotometer (Thermo). Expression of miR‐141 was assayed using stem‐loop RT followed by qRT‐PCR analysis 15. Reagents for stem‐loop RT and qRT‐PCR were obtained from Thermo Scientific. qRT‐PCR was performed in triplicate and the results were calculated using the 2^−ΔCT^ method 16, where ΔCt = Ct _miR‐141_ ‐ Ct _U6_. Similarly, the expression of *ANP32E* mRNA in transfected cells was examined by qRT‐PCR in triplicate and calculated using the 2^−ΔΔCT^ method, where ΔΔCt = ΔCt _miR‐141 group_‐ΔCt _NC group,_ ΔCt = Ct _ANP32E_‐Ct _*β*‐actin_.

### Western blot analysis

MDA‐MB‐231 cells were inoculated into 6‐well culture plates at 3 × 10^5^ cells per well. Forty eight h after transfection, Western blot analysis was performed to measure the ANP32E protein expression levels in the cells. Each well of the culture plates was lysed in 1 mL of Radio‐Immuno precipitation Assay (RIPA) lysis buffer: 150 mmol/L NaCl, 1% NP40, 0.5% sodium deoxycholate, 0.1% SDS, 50 mmol/L Tris (pH 7.9), 10 mmol/L NaF, 10 mmol/L PMSF, and 1 ×  protease inhibitors (Roche). The protein concentration was measured using a BCA method. The relative expression level of ANP32E was represented by ANP32E/*β*‐actin ratio 17. The following antibodies were used: ANP32E polyclonal antibody and mouse anti‐human *β*‐actin monoclonal antibody (Abcam).

### miRNA target prediction

miRNA target prediction data from three databases were used for miR‐141 target prediction. The three target prediction databases included microRNA.org (http://www.microrna.org/microrna/getGeneForm.do), Targetscan (http://www.targetscan.org/vert_71/), and PITA (https://genie.weizmann.ac.il/pubs/mir07/mir07_data.html). In all 37 shared putative miR‐141 targets were identified from the three databases. Based on gene functional relevance, *ANP32E* was chosen for further validation.

### Luciferase reporter assays

The 3′ UTR sequences of the *ANP32E* gene were amplified from the genomic DNA of normal human surrounding tissues and subcloned directly downstream of the *Renilla* luciferase gene of a psiCHECK2 vector. Primer sequences used to amplify this region were *ANP32E* 3′UTR‐F: 5′‐CCGCTCGAGATCATTCTAAGACCAGATTCTCTAA‐3′ and *ANP32E* 3′UTR‐R: 5′‐ ATTTGCGGCCGCCAAATCTTCAATTTATTTGAAGCAATTCAG‐3′. A mutant version of *ANP32E* within the “seed region” was generated using the QuickChange II Site‐Directed Mutagenesis Kit (Stratagene). All the constructs were verified by DNA sequencing. MDA‐MB‐231 cells were seeded in 96‐well plates at a density of 5000 cells per well. After 24 h, cells were cotransfected with 25 pmol of miR‐141 mimics or NC mimics, along with 100 ng per well of psiCHECK2‐3′UTR‐ANP32E construct or psiCHECK2‐3′UTR‐ANP32E mutant using Lipofectamine^TM^ 2000 according to the manufacturer's protocol. Cells were grown for 48 h, Firefly and *Renilla* luciferase activities were quantified using the Dual‐Luciferase Reporter Assay System (Promega). The ratio of *Renilla* luciferase/Firefly luciferase was calculated as the reporter activity.

### Construction of ANP32E‐RNAi lentiviral vectors, cell transfection, and analysis of cell function

The *ANP32E* RNAi lentiviral vectors (LVpGCSIL‐puro‐shRNA) were constructed by Shanghai GeneChem Co, Ltd. A lentiviral vector (LVpGCSIL‐puro) was used as a negative control. The targeted sequences of VshRNA #1, #2, and #3 are 5′ TCTCATACTTAATGAAAGA 3′, 5′ ATGGCTAATGTGGAACTAA 3′, and 5′ AGCTTAAATAAACTTCGAA 3′, respectively. MDA‐MB‐231 cells were infected with LVpGCSIL‐puro‐shRNA or LVpGCSIL‐puro at the MOI of 10. Cells were selected with puromycin 72 h after infection, and cells were used for functional studies 48 h after selection. qPCR was used to screen for the most efficient vshRNA for subsequent study. Cell proliferation, migration, and matrigel invasion assays were conducted as described above. Migration assays were performed similar to the invasion transwell assays without the matrigel coating.

### Statistical analysis

Statistical differences between groups were determined by Student's *t* test. Statistical differences among groups were determined by ANOVA. Data are presented as mean ± standard deviation (M ± SD). *P *<* *0.05 was considered to be statistically significant.

## Results

### Downregulation of miR‐141 in BC tissues and its correlation with clinicopathologic parameters

**Figure 1:**
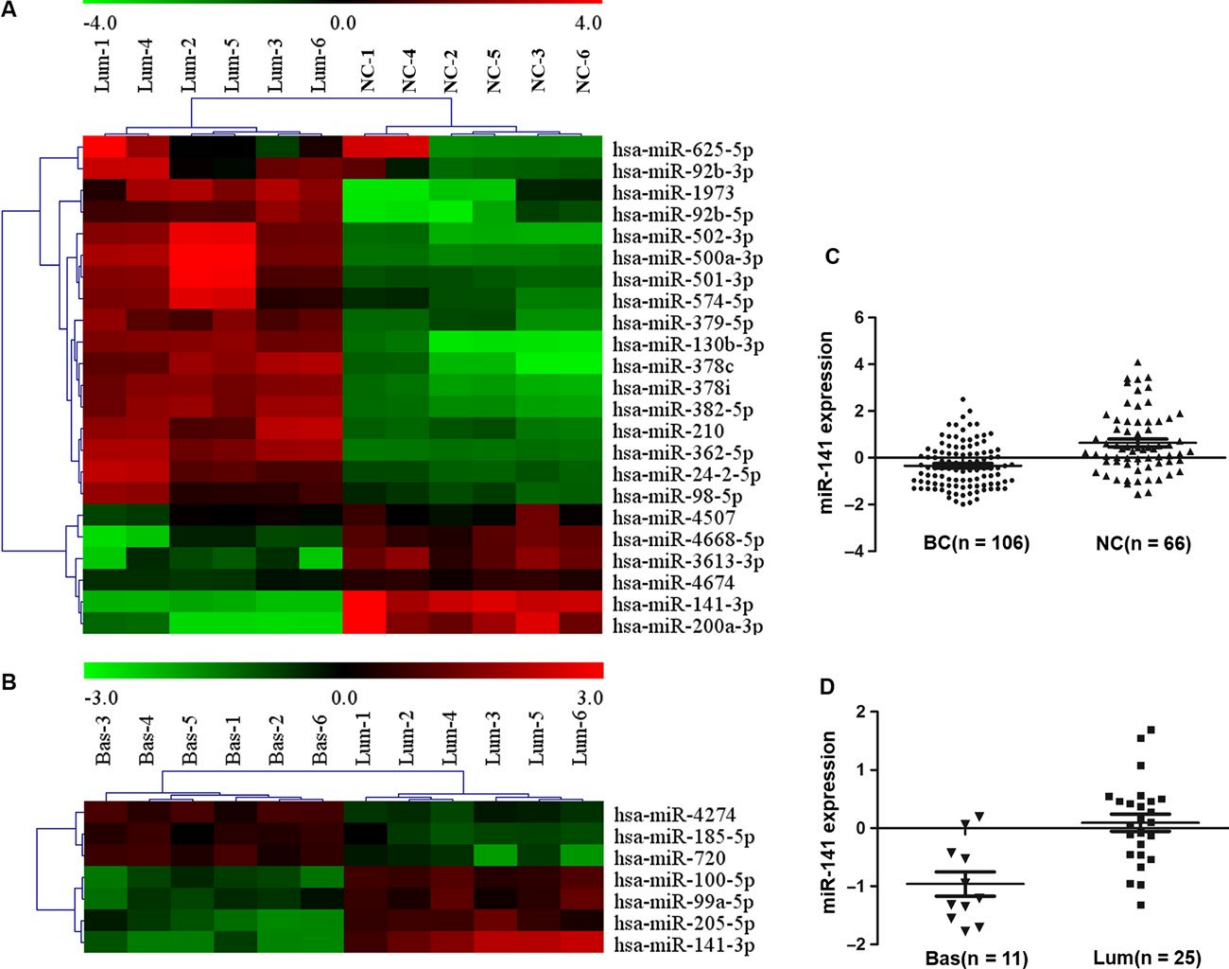
Differential expression of miRNAs in breast cancer tissues. (A) and (B) Microarray analysis data showing the relative expression of miR‐141 in breast cancer (BC) tissues. Color gradation indicates the relative expression level of miRNAs from low expression (green) to high expression (red). (C) and (D) qPCR analysis data showing the relative expression of miR‐141 in BC tissues. (A) and (C) miR‐141 was downregulated in BC tissues with luminal‐like subtype (Lum) compared with surrounding tissues (NC,* P *<* *0.001). (B) and (D) miR‐141 was downregulated in BC tissues with Basal‐like subtype (Bas) compared with Lum tissues (*P *<* *0.001).

To identify miRNAs that may play important roles in BC development, two independent miRNA microarray assays were performed. In the first experiment, six BC FFPE specimen of Luminal A&B subtype and with ER, PR, and HER‐2 positive (“Lum”) were used, together with six paired nonmalignant tissues (surrounding tissues, normal control, NC). The results showed that 17 miRNAs were significantly upregulated and six were significantly downregulated (Fig. 1A). In the second experiment, six “Lum” tissue specimen and six BC specimen of Basal‐like subtype, and with ER, PR, and HER‐2 all negative (“Bas”) were compared with. As shown in Figure 1B, miR‐205, miR‐100, miR‐99a, and miR‐141 were downregulated in BC‐Bas tissues. Downregulation of miR‐141 was observed in both rounds of microarray assays, and miR‐141 was selected for further studies. qRT‐PCR was performed to measure the levels of miR‐141 in 106 BC FFPE tissues and 66 surrounding tissues, as well as in 11 BC‐Bas tissues and 25 BC‐Lum tissues. Consistent with the results from miRNA microarray assays, the downregulation of the miR‐141 was observed in BC FFPE tissues compared with surrounding tissues (Fig. 1C), and miR‐141 expression was lower in BC‐Bas tissues than in BC‐Lum tissues. (Fig. 1D).

Furthermore, miR‐141 expression level in BC tissues correlated negatively with tumor stage, lymph node metastasis, and expression levels of HER2, PCNA, and Ki67 (Table 1), while there was no significant correlation between miR‐141 expression and patient age, tumor size, tumor grade, ER, or PR expression levels (data not shown).

### Effect of miR‐141 overexpression on cell functions

**Figure 2:**
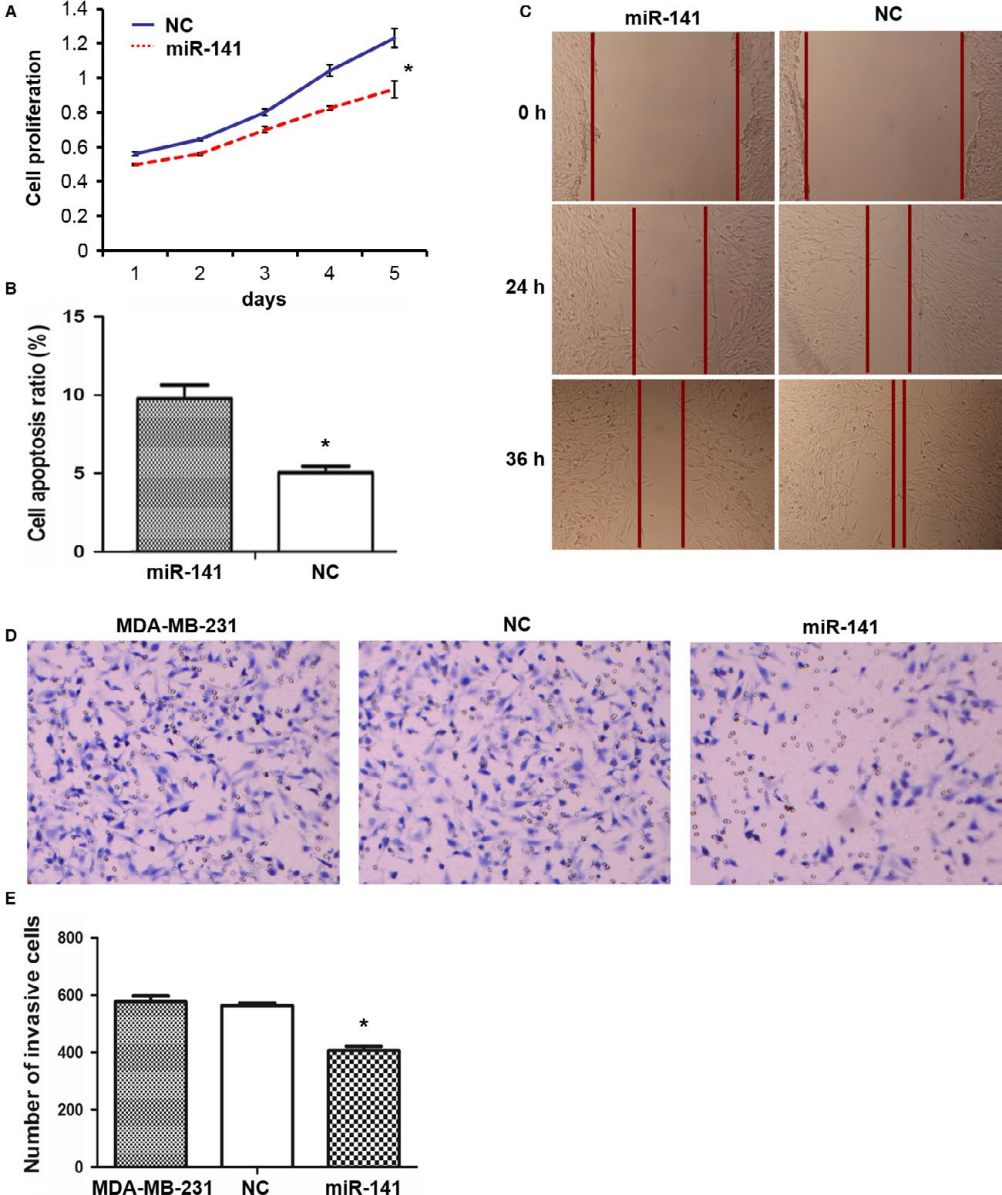
miR‐141 overexpression inhibits cell proliferation, enhances apoptosis, and inhibits cell invasion and migration. MDA‐MB‐231 cells were transfected with miR‐141 mimics or NC mimics, and cell functional studies were performed. Results represent Means and SD of three experiments except *C* where a representative set of figures of multiple experiments was shown. *indicates statistical significance. (A) Cell proliferation by CCK‐8 assays. (B) Apoptosis by Annexin V‐FITC/PI staining and flow cytometry analysis. (C) Cell migration by monolayer cell wound assays. (D) and (E) Cell invasion by matrigel transwell assays.

CCK‐8 assays were performed to examine the effect of miR‐141 on cell proliferation. Cell proliferation in the miR‐141 mimics transfected MDA‐MB‐231 cells was poorer than that of the NC mimics group, suggesting that overexpression of miR‐141 affected MDA‐MB‐231 cell proliferation (Fig. 2A). Forty eight hours after MDA‐MB‐231 cells were transfected with miR‐141 mimics or NC mimics, cells were stained with Annexin V‐FITC and PI, and apoptosis were measured by flow cytometry. Transfection of MDA‐MB‐231 cells with miR‐141 mimics caused significant more apoptosis than the NC group (Fig. 2B, Fig. S1*a*).

Wound healing assays were performed to examine the effect of miR‐141 on MDA‐MB‐231 cell migration. As shown in Figure 2C and Fig. S1*b*, the cell‐free scratched area of the miR‐141 transfected group was significantly wider than the NC group at 24 h and at 36 h after scratching on the monolayer cells, indicating significantly less cell migration with miR‐141 overexpression. Transwell migration assays were performed to investigate the role of miR‐141 on MDA‐MB‐231 cell invasion. As shown in Figs. 2D and E, the number of invaded cells was significantly less in the miR‐141 transfected group compared with NC group.

### ANP32E as a target of miR‐141

**Figure 3:**
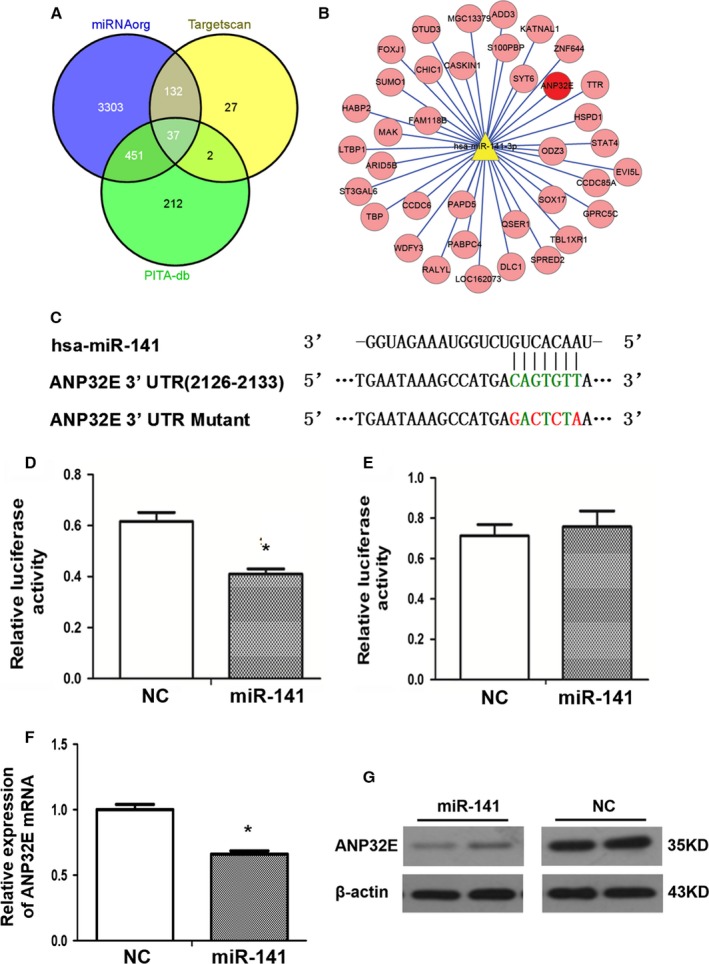
miR‐141 targets *ANP32E* in cells. (A) Venn diagram of miR‐141 target gene prediction results from three databases. (B) *ANP32E* (red) was one of 37 shared predicted target genes**.** (C) The putative miR‐141‐binding site in the 3′UTR sequence of the gene *ANP32E* and mutation positions. (D) and (E) Luciferase activity assays of MDA‐MB‐231 cells cotransfected with a wild‐type (D) or a mutant (E) *ANP32E* 3′UTR reporter construct and miR‐141 mimics or NC mimics. Results represent Means and SD of three experiments. *indicates statistical significance. (F) The mRNA levels of *ANP32E* in MDA‐MB‐231 cells after transfection with miR‐141 mimics or NC mimics analyzed by qRT‐PCR. Results represent Means and SD of three experiments. *indicates statistical significance. (G) ANP32E protein levels in MDA‐MB‐231 cells after transfection with miR‐141 mimics or NC mimics analyzed by Western blot analysis. Samples were loaded in duplicate.

miRNAs usually play important roles in cellular functions by targeting critical downstream genes. miRNA target prediction data from three databases were used for miR‐141 target prediction. The three target prediction databases included microRNA.org, Targetscan, and PITA. In all 37 shared putative miR‐141 targets were identified from the three databases. Judged from gene functional relevance, *ANP32E* was chosen for further validation (Figs. 3A and B). The Targetscan program identified one conserved binding site for miR‐141 in the 3′UTR region of the *ANP32E*, which was perfectly complementary to the 2–8 nt of the miR‐141 (Fig. 3C).

A significant decrease in relative luciferase activity was observed when psiCHECK2‐ ANP32E 3′UTR was cotransfected with miR‐141 mimics in MDA‐MB‐231 cells as compared with the NC mimics (*P *<* *0.05, Fig. 3D). When a mutant version of psiCHECK2‐ANP32E 3′UTR with a 7‐bp mutation within the seed region was used in the cotransfection assays, no significant difference was identified between miR‐141 mimics group and NC group (Fig. 3E).

The mRNA level of *ANP32E* as analyzed by qRT‐PCR decreased significantly in the miR‐141 mimics transfected group than the NC group (Fig. 3F). Western blot analysis showed that ANP32E protein levels in the miR‐141 mimics transfected group were significantly lower than that in the NC group (Fig. 3G).

### Effect of direct ANP32E knockdown on cell proliferation, migration, and invasion

**Figure 4:**
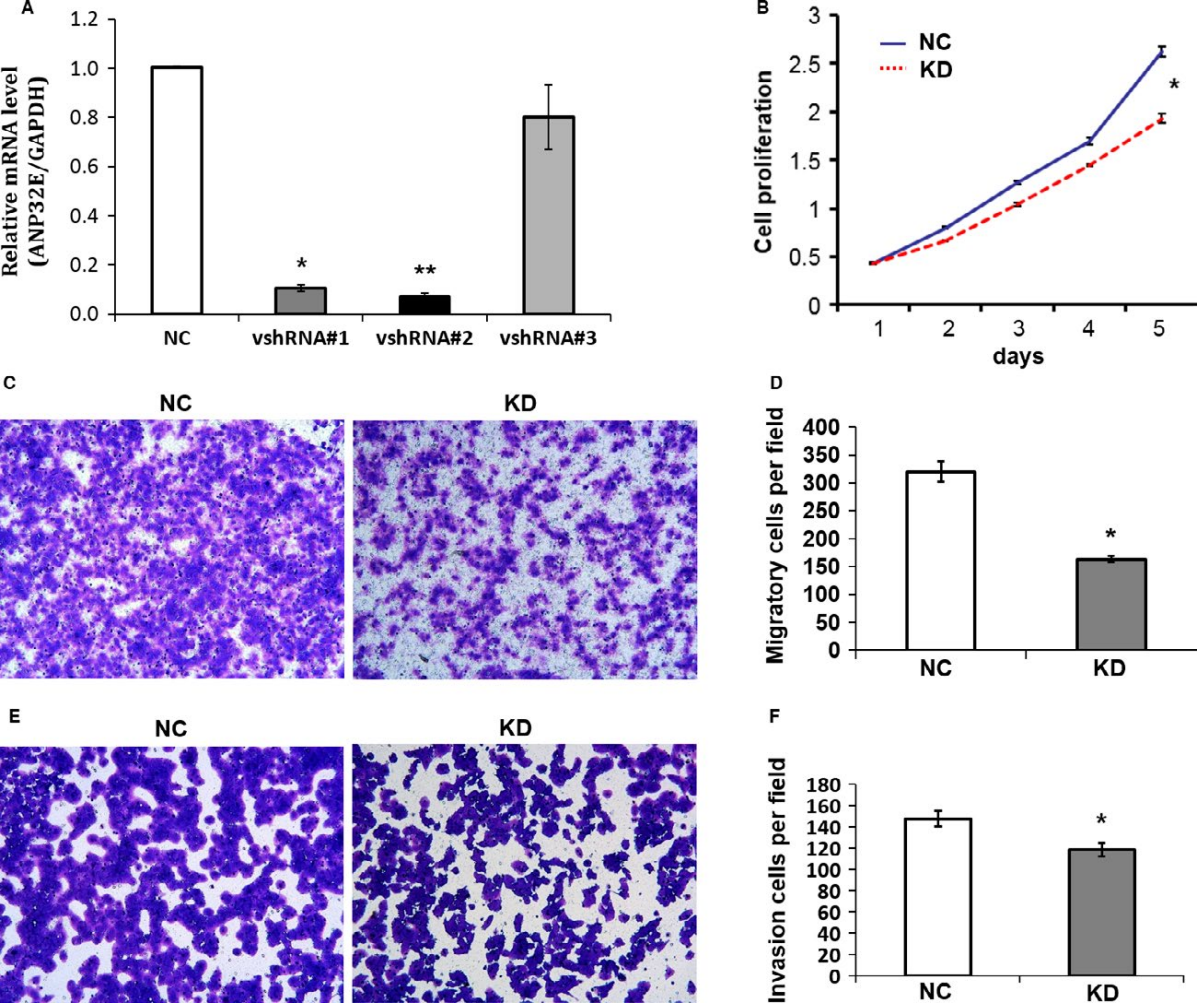
Knockdown of *ANP32E* in cells decreased cell proliferation, migration, and invasion. MDA‐MB‐231 cells were infected with LVpGCSIL‐puro (NC group) and LVpGCSIL‐puro‐shRNA (KD group), respectively. Infected cells were selected with puromycin and used for functional assays. Results represent Means and SD of three experiments. *indicates statistical significance. (A) The efficiency of ANP32E knockdown was verified by qPCR. (B) Cell proliferation by CCK‐8 assays. (C) and (D) Cell migration by uncoated transwell assays.(E) and (F*)* Cell invasion by matrigel‐coated transwell assays.

*ANP32E* expression in MDA‐MB‐231 cells was knocked down using specific vshRNAs to investigate the effect of ANP32E on cell proliferation, migration, and invasion. Among the three candidate shRNAs tested, shRNA#2 was the most efficient, and the efficiency of knockdown was 93.1% (Fig. 4A). MDA‐MB‐231 cells were infected by RNAi lentivirus (LVpGCSIL‐puro‐shRNA), selected by puromycin, and used for functional assays. As compared with vector control group (NC), cells in the ANP32E knockdown group (KD) showed significant less proliferation, migration, and invasion (Fig. 4B–F).

## Discussion

miRNAs are key regulators of various fundamental biological processes, such as cancer initiation and progression, sustained proliferative signaling, resisting cell death, activating cell migration and invasion, inducing angiogenesis, avoiding immune destruction or deregulating cell energetics. The investigation of miRNAs associated with aggressive behavior in BC may provide prognostic and disease monitoring markers and potentially define novel therapeutic targets 18, 19. MiR‐141 belongs to the miR‐200 family, which consists of the following members: miR‐141, miR‐200a, miR‐200b, miR‐200c, and miR‐429 20. Overexpression of hsa‐miR‐141 has been shown to inhibit invasion and migration of breast cancer, colorectal cancer, and pancreatic cancer 10, 21, 22. miR‐141 was also found to regulate cancer cell growth and metastasis in lung cancer, liver cancer, colorectal cancer, gastric cancer, renal cancer, and prostate cancer 8, 9, 10, 11, 12, 13. In this study, we found that miR‐141 was downregulated in breast cancer in tumor tissues compared with matched surrounding tissues. Downregulation of miR‐141 expression correlated with tumor stage, lymph node involvement, and expressions of PCNA, Ki67, and HER2. Moreover, overexpression of miR‐141 inhibited BC cell proliferation, migration and invasion in vitro. The expression levels of miR‐141 correlated negatively with HER2 expression in BC tissues in our study, raising an interesting question as how the two pathways converge to regulate BC growth and development.

In an attempt to search for potential targets of miR‐141, we came across with *ANP32E*. ANP32E is a histone chaperone that removes H2A.Z from chromatin, and promotes nucleosome reorganization and DNA repair 23, 24. *ANP32E*‐deficient mice are viable and show no obvious abnormal phenotypes 25. *ANP32E* was identified as one of the six‐member predictors for BC invasion and metastasis 26. ANP32E protein was also found to be downregulated in the cytoplasm of mouse fibrosarcoma cells 27. Considering the significant roles of miR‐141 in inhibiting cell migration and invasion in different cancer types, and the potentially significant effect of *ANP32E* in cancer cells, we reason that miR‐141 and *ANP32E* might have some direct interaction in BC cells. Indeed, results from luciferase reporter assays shows that miR‐141 directly inhibited *ANP32E* transcriptions, and overexpression of miR‐141 inhibited *ANP32E* expression at both mRNA and protein levels. Moreover, knockdown of *ANP32E* by siRNA lentivirus inhibited BC cell proliferation, migration and invasion, mimicking the effect of overexpression of miR‐141 in vitro. Our data suggest that while miR‐141 might function as a tumor suppressor, ANP32E functions as a positive regulator of tumor growth and metastasis.

How *ANP32E* regulates BC cell migration and invasion? Little is known about ANP32E's functions inside cells except that it is a histone chaperone that removes H2A.Z from chromatin, and it promotes nucleosome reorganization. Epithelial–mesenchymal transition (EMT) is a process in which epithelial cells lose their cell–cell adhesion, and acquire migratory and invasive properties as mesenchymal cells. EMT is a process involved in embryogenesis and wound healing, and is considered to be critical in mediating cancer metastasis. Several pathways, including TGF‐β pathways, Notch pathways, and Wnt pathways, converge at SNAIL which regulates EMT process, and these pathways are involved in the regulation of cancer metastasis 28, 29, 30. Where to put *ANP32E* in cancer metastasis regulation network remains unknown at this time. Chromatin remodeler proteins function as gatekeepers and constitute a major determinant of accessibility of cellular factors to nucleosome DNA, thus regulating many cellular activities. Aberrant expression or epigenetic modulation of remodeler proteins confers a unique ability to cancer cells to reprogram its genome for growth or metastasis 31. Salz et al. reported that histone methyltransferase hSETD1A was a novel regulator of BC metastasis 32. Lysine histone demethylase KDM2A was also reported to be a regulator of BC invasion and metastasis 33. The nonhistone chromatin‐binding protein HMGA2 was found to be a driver of cancer metastasis by targeting TGF‐β 34. PELP, a nuclear receptor coregulator, was also found to regulate EMT and BC metastasis by controlling the expression of miR‐141 35. Metastasis‐associated tumor antigen 1 (MTA1) is a chromatin modifier, and an integral part of nucleosome remodeling and histone deacetylation (NuRD) complex 36. Elucidation of the mechanism of *ANP32E* involved in BC invasion and metastasis would enhance our understanding of the complex cellular process, and provide clues to better therapeutic intervention.

Results from our study add our knowledge on the roles miR‐141 in BC development and metastasis as well as shed more light on the roles a new player *ANP32E* in the processes. Our study helps the understanding of complex signaling network in BC development, progression, and metastasis. Both *ANP32E* and miR‐141 might serve as prognosis predictive factors and potential therapeutic targets in BC diagnosis and treatment.

## Conflicts of Interest

The authors declare no conflict of interests.

## Supporting information


**Figure S1*a*.** Apoptosis analysis of MDA‐MB‐231 cells transfected with miR‐141 mimics or NC mimics. Analyzed data were shown in Figure 2B.

**Figure S1*b*.** Quantification of cell migration in wound healing assays as described in Figure 2C. Cell images were analyzed with Photoshop. The mean gap width in NC group at 0 time point was arbitrarily set as 100%.
