# Supplementary figures and images for: Downregulation of miRNA‐141 in breast cancer cells is associated with cell migration and invasion: involvement of ANP32E targeting
Source: Cancer Med. 2017 Feb 21;6(3):662–72. doi: 10.1002/cam4.1024 (PMC5345683; doi:10.1002/cam4.1024)

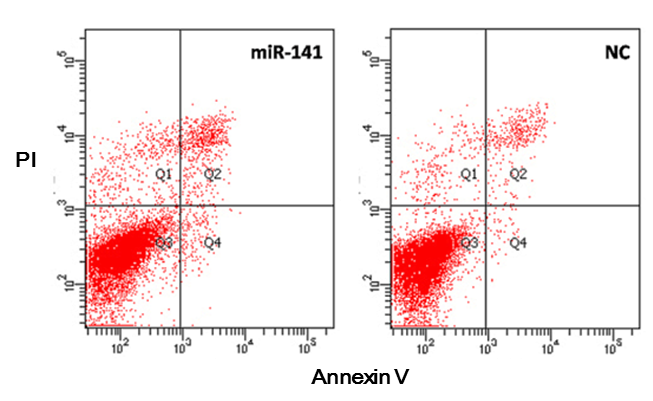

Supplement: Supplementary file 1 — Figure S1a. Apoptosis analysis of MDA‐MB‐231 cells transfected with miR‐141 mimics or NC mimics. Analyzed data were shown in Figure 2B. [file CAM4-6-662-s001.tif]

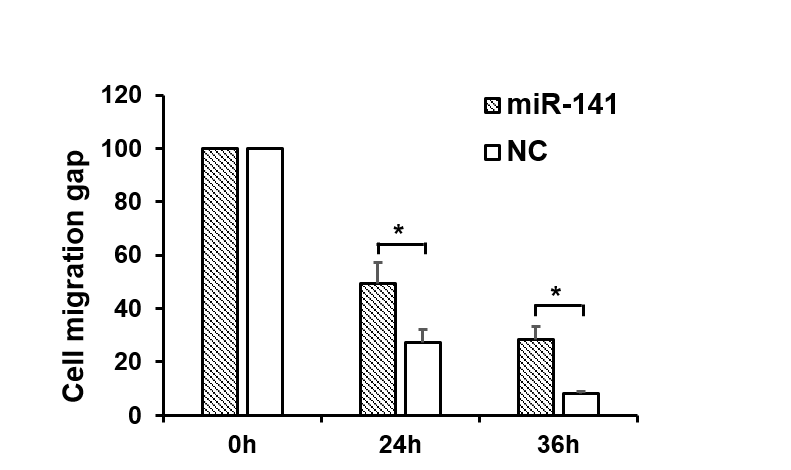

Supplement: Supplementary file 2 — Figure S1b. Quantification of cell migration in wound healing assays as described in Figure 2C. Cell images were analyzed with Photoshop. The mean gap width in NC group at 0 time point was arbitrarily set as 100%. [file CAM4-6-662-s002.tif]
